# Development of infectious cDNA clones of Salmonid alphavirus subtype 3

**DOI:** 10.1186/1756-0500-3-241

**Published:** 2010-09-21

**Authors:** Marius Karlsen, Stephane Villoing, Karl F Ottem, Espen Rimstad, Are Nylund

**Affiliations:** 1 Department of Biology, University of Bergen, Thor Møhlens gate 55, 5020 Bergen, Norway; 2 Intervet Norbio, Thor Møhlens gate 55, 5008 Bergen, Norway; 3 Norwegian School of Veterinary Science, Oslo, Norway

## Abstract

**Background:**

Salmonid alphavirus (SAV) is a widespread pathogen in European aquaculture of salmonid fish. Distinct viral subtypes have been suggested based on sequence comparisons and some of these have different geographical distributions. In Norway, only SAV subtype 3 have so far been identified. Little is known about viral mechanisms important for pathogenesis and transmission. Tools for detailed exploration of SAV genomes are therefore needed.

**Results:**

Infectious cDNA clones in which a genome of subtype 3 SAV is under the control of a CMV promoter were constructed. The clones were designed to express proteins that are putatively identical to those previously reported for the SAVH20/03 strain. A polyclonal antiserum was raised against a part of the E2 glycoprotein in order to detect expression of the subgenomic open reading frame (ORF) encoding structural viral proteins. Transfection of the cDNA clone revealed the expression of the E2 protein by IFAT, and in serial passages of the supernatant the presence of infectious recombinant virus was confirmed through RT-PCR, IFAT and the development of a cytopathic effect similar to that seen during infection with wild type SAV. Confirmation that the recovered virus originated from the infectious plasmid was done by sequence identification of an introduced genetic tag. The recombinant virus was infectious also when an additional ORF encoding an EGFP reporter gene under the control of a second subgenomic alphavirus promoter was added. Finally, we used the system to study the effect of selected point mutations on infectivity in Chinook salmon embryo cells. While introduced mutations in nsP2_197_, nsP3_263 _and nsP3_323 _severely reduced infectivity, a serine to proline mutation in E2_206 _appeared to enhance the virus titer production.

**Conclusion:**

We have constructed infectious clones for SAV based on a subtype 3 genome. The clones may serve as a platform for further functional studies.

## Background

The genus *Alphavirus *(Family *Togaviridae*) consists of viruses with positive sense, single stranded, capped and polyadenylated RNA genomes [1]. Of the 29 recognized species in the genus, 28 are pathogens of higher vertebrates in the terrestrial environment, and the transmission cycle of these viruses includes an arthropode vector. The only species of the genus that infects fish, Salmon pancreas disease virus, today more commonly designated Salmonid alphavirus (SAV), is genetically distinct and a pathogen of farmed salmonids in Europe [2-4]. The genome of SAV is 11.9 kb long and has a genomic structure homologous to terrestrial alphaviruses with two large open reading frames (ORFs) of 8 and 4 kb length that are flanked by three untranslated regions (UTRs). The first ORF encodes non-structural proteins 1-4 (nsP1-4) and the second one encodes the structural proteins capsid, E3, E2, 6K, TF and E1 [5,6], of which capsid, E2 and E1 have been demonstrated to be expressed during replication [7,8]. The nsPs, possibly together with host proteins, make up the replicase complex (RC) that replicates the viral genome and transcribes the second ORF [9,10]. The structural proteins are translated from the subgenomic mRNA of the second ORF, that is controlled by a SAV RC specific promoter [2,3,10]. Following its translation in the cytoplasm, the capsid protein cleaves itself from the adjacent structural proteins. Studies from terrestrial alphaviruses have demonstrated that the cleaved capsid interacts with viral genomic RNA to form nucleocapsids [1]. It has also been suggested to have additional non-structural functions as the capsid of several alphaviruses, including SAV, may localize to the nucleus during infection [8,11-15]. The remaining structural proteins are translated into the membrane of the endoplasmatic reticulum, where they undergo glycosylation and proteolytic cleavage, before they are transported to the cell membrane. Budding of viral particles is induced by interactions between the capsid protein and the cytosolic parts of viral glycoproteins [16]. The glycoproteins are functional in the recognition and binding to receptors on the cell surface (E2), and fusion of the viral membrane with the host cellular membrane (E1) [1]. It is likely that SAV uses the same route of budding, entry and glycoprotein maturation, based on homology in sequence motifs between alphaviruses and the observed intracellular localization of the glycoproteins during infection [2,3,7].

The SAV replication cycle can be reproduced and modified using reverse genetics systems [10]. In these systems the complete or partial viral genome is cloned as cDNA where a promoter for RNA transcription controls the expression. Such systems may be designed as replicons, where the viral structural ORF is replaced with a gene of interest (GOI). Alternatively, the structural ORF is intact and addition of a second alphaviral promoter controls expression of the GOI. Expression of the GOI will then follow the same kinetics as the viral structural proteins.

Sequence analyses of SAV strains have suggested that at least six genetically distinct subtypes of the virus, SAV1-6, have evolved [17]. Some of the observed sequence differences also lead to variations in antigenic epitopes [5]. Of these subtypes, SAV1, 2 and 3 are the best studied, and full-length genomes of strains belonging to these subtypes have been sequenced [5,9,18]. Strains grouping to subtype 1 have primarily been associated with disease in the marine production phase of Atlantic salmon (*Salmo salar*) in Ireland, those grouping to subtype 2 with disease in Rainbow trout (*Oncorhynchus mykiss*) in the freshwater phase in continental Europe and UK, and recently reported from marine Atlantic salmon in Scotland, and subtype 3 strains with disease in Atlantic salmon and Rainbow trout in the marine production phase in Norway [17-19]. Experimental infection trials have demonstrated that strains belonging to the different subtypes cause similar clinical signs and pathologies in the target species [4]. With the exception of SAV2 infections in rainbow trout fingerlings [10], the mortality rates observed in laboratory experimental transmission of SAV are typically low, if any mortality at all [5,20-22]. Higher mortalities have sometimes been reported from field outbreaks [23,24]. Although these variations in mortality could be explained by differences in virulence between viral strains, the contribution from factors other than SAV, such as fish strain, environmental variations and other pathogens, could be equally important. Different transmission routes of SAV have been suggested to be of importance in aquaculture [17,25-28], but transmission mechanisms remain poorly understood. In Norway, SAV appears to be genetically homogenous, suggesting one or few original common sources for all outbreaks [25]. After an outbreak, SAV may remain in the fish population in the form of a possibly lifelong, persistent infection in some individuals [20,29,30]. Information about viral and host mechanisms that regulate the persistency is not available, but could be useful for proper management of infected fish populations.

Although viral factors that contribute to the different outcomes of a SAV infection remain largely unresolved, we previously hypothesised based on sequence analyses that a mutation in E2, the viral receptor protein, could be of importance for pathogenicity [25]. In general, outbreaks of several alphaviral epidemics have been associated with such point-mutations in E2 and E1 [31,32]. Recently, studies of the epidemic caused by the alphavirus Chikungunya virus (CHIKV), have demonstrated how reverse genetics systems can be powerful tools for understanding evolution, mechanisms of pathogenesis and genetic factors contributing to epidemics [32-34]. Reverse genetics systems addressing such topics should express viral proteins that are closely related to those of the naturally occurring virus. A reverse genetics system has been developed for an attenuated strain of SAV2 originating from rainbow trout [10]. Here we report the construction of a reverse genetics system that enables the recovery of a recombinant SAV subtype 3 with putatively identical proteins to those previously reported from the SAVH20/03 strain originally isolated from diseased Atlantic salmon in Norway [18]. The system can be particularly useful for studies of evolution and mechanisms of pathogenesis and transmission of SAV.

## Results

### Detection of the E2 glycoprotein in infected cells

**Figure 1:**
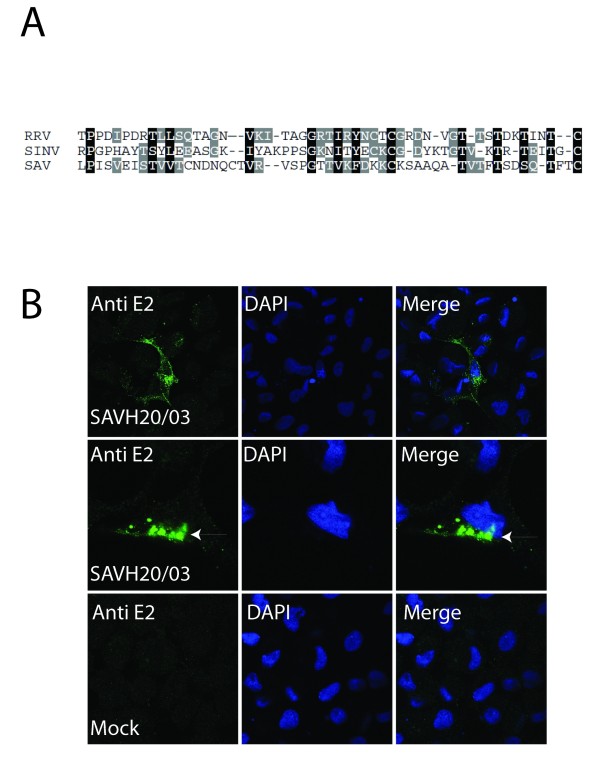
**Development of polyclonal antiserum against E2 and detection of SAVH20/03 by IFAT**. (A) A region of E2 was identified based on alignment of SAV3 with antigenic regions of the SINV and RRV E2 proteins. The peptide corresponded to amino acids 184 to 236 of the putative SAV3 E2 protein was cloned and expressed in *E. coli*, and used for immunization of a rabbit. (B) IFAT using the obtained E2 antiserum on CHSE cells infected with SAVH20/03 demonstrated specific staining located to the plasma membrane and cytoplasmic structures, possibly ER and Golgi (arrow). No staining could be observed in mock treated cells.

Proper tools for detection of structural proteins from SAV3 strains are essential for confirmation of a functional reverse genetics system. We therefore obtained an antiserum against the E2 protein. A partial E2 coding sequence of SAVH20/03 was chosen based on a nucleotide alignment with a known antigenic region in Sindbis virus (SINV) and Ross River virus (RRV) (Figure 1a). The fragment was successfully expressed as a His-tagged peptide in E. coli (results not shown) and used to immunize a rabbit. Specificity of the obtained antiserum was verified by IFAT on Chinook salmon embryo (CHSE) cell cultures infected with the wild type SAV3 strain SAVH20/03. A positive signal was localized to the cell membrane (Figure 1b) and to cytoplasmic structures believed to be ER and Golgi (Figure 1b, arrow) as visualized by confocal microscopy. This corresponds well with the previously reported localization of E2 during infections with SAV [7,35], as well as with the localization of E2 of terrestrial alphaviruses [16]. No staining could be observed in mock treated cells (Figure 1b).

### Construction of a full-length SAV3 cDNA clone

**Figure 2:**
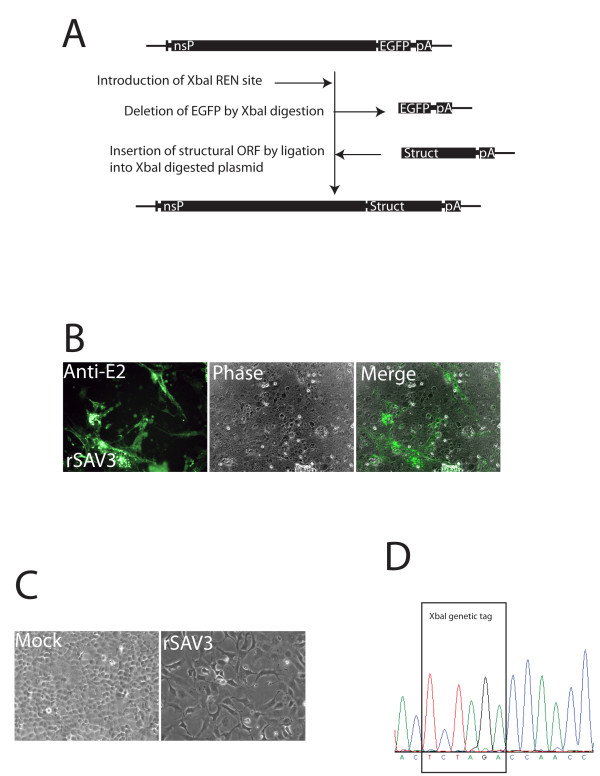
**Construction of a full-length infectious SAV3 cDNA and recovery of infectious virus**. (A) A cDNA fragment containing the EGFP ORF and 3'UTR of a modified pmSAV3 plasmid was exchanged with the structural ORF and 3'UTR through an introduced XbaI site in the junction region. (B) IFAT on CHSE cells infected with supernatant from cells transfected with the prSAV3 plasmid. (C) Cytopathic effect in CHSE cells infected with supernatant containing recombinant SAV3 passage 3. (D) Identification of the introduced XbaI genetic tag by sequencing of an RT-PCR product obtained from RNA extracted from infected cell layers (passage 3) showing cytopathic effect.

We have recently reported the construction of the DNA replicon-construct pmSAV3, where a SAV3 RC expresses an EGFP ORF from an alphavirus promoter [9]. In order to obtain a full-length infectious clone, we replaced the EGFP ORF with the structural ORF of SAV through an introduced XbaI site (Figure 2a). The XbaI site was kept in the final construct and functioned as a genetic tag that could be used to verify the origin of progeny virus from infectious clones. Sequencing of the obtained full-length cDNA clone, prSAV3, revealed amino acid sequence identity, but 10 nucleotide mutations, not leading to amino acid changes, were present compared to previously reported consensus sequences of SAVH20/03 (accession nos. AY604235 and DQ149204). Of these, four were located in the non-structural ORF, two constituted the genetic tag in the junction, two were located in E2 and two were located in the 3'UTR (Table 1). Selection of a proper *E. coli *strain (XL10 Gold, Stratagene) was crucial for the propagation of these plasmids since proliferation of *E. coli *strain Top10 (Invitrogen) was dramatically reduced after transformation with plasmids harbouring the full-length sequence.

**Table 1:** Nucleotide differences between prSAV and SAVH20/03 (positions refer to accession no. AY604236).

Position	Region	Mutation
2732	nsP2	A->G
4098^1^	nsP2	A->G
5427	nsP3	C->T
7539	nsP4	A->G
7548	nsP4	A->G
7737-7738	Junction	CA->AG
8867	E2	T->C
9278	E2	G->A
83^2^	3'UTR	->T
97^2^	3'UTR	A->T

^1^This position has previously been reported to be a G (accession no. DQ149204) or an A (accession no. AY604236) in SAVH20/03.

^2^The complete 3'UTR is not reported in AY604236 and positions are given relative to the 3'UTR start nucleotide [9].

### Recovery of infectious recombinant SAV from cDNA

The ability of the full-length clone to express viral structural proteins was addressed using the obtained antiserum against E2. IFAT with anti-E2 on transfected Blue gill fry 2 (BF2) and CHSE cells demonstrated a positive signal 12 days post transfection (d.p.t.) similar to that seen during infection with wild type SAV (not shown). Passage of supernatant from transfected cells onto non-infected CHSE and BF2 cells followed by IFAT on these, confirmed presence of infectious particles in the supernatant (Figure 2b). Following 3 passages in CHSE cells, a cytopathic effect similar to that of wild type SAV infection was evident (Figure 2c). The CPE included elongated, vacuolated cells with pseudopodia-like extensions and curled up, dead cells. The authenticity of the recombinant virus (rSAV3) was verified by RT-PCR and sequencing on passage 3 virus of the unique XbaI site that had been introduced in the junction region of the rSAV3 genome (Figure 2d).

### Recovery of infectious transgenic SAV with two subgenomic promoters

**Figure 3:**
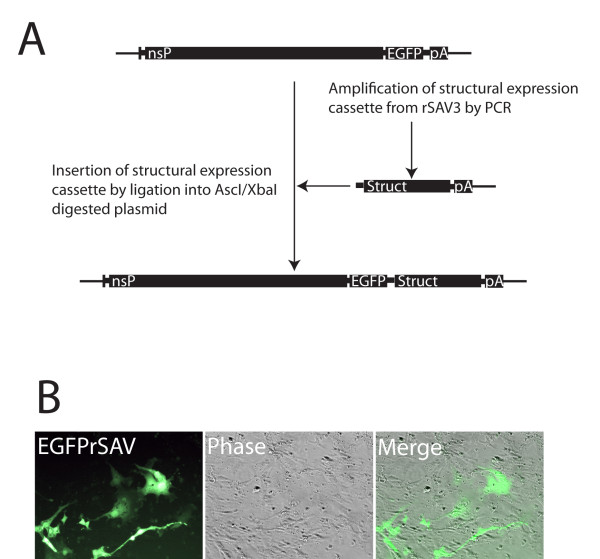
**Construction and validation of recombinant SAV3 with a double subgenomic promoter**. (A) The full-length SAV3 was engineered to contain an additional ORF encoding the EGFP protein under the control of a SAV specific promoter. The construct was obtained by AscI/NotI cleavage and ligation of a PCR amplified fragment encoding SAV3 structural proteins and the SAV3 subgenomic promoter, into a modified pmSAV3 plasmid. (B) The obtained construct was transfected into BF2 cells and expression of EGFP was visualized by fluorescence microscopy. Presence of infectious virus containing the EGFP ORF was verified by serial passage of supernatant from transfected cells onto naïve CHSE cells followed by fluorescence microscopy of live cells.

A recombinant infectious virus with two subgenomic promoters was obtained by introducing the structural polyprotein ORF of SAV into a modified pmSAV3 replicon plasmid, making the final pEGFPrSAV3 plasmid (Figure 3a). The sequence of pEGFPrSAV3 was identical to that of prSAV3 with the exception of the additional subgenomic cassette expressing EGFP from the subgenomic promoter of SAV located upstream of a transcription cassette expressing the structural ORF. The exact start of the subgenomic promoter in the nsP4 sequence is not known for SAV, but the length of the fragment chosen was based on a previously reported SAV2 promoter that had been shown to be functional [10]. The EGFP ORF is flanked by AgeI and AscI sites that allow exchange of the EGFP ORF with other cDNAs. Transfection of pEGFPrSAV3 into BF2 cells produced EGFP expression 5 days p.t. Foci of cells expressing EGFP were evident and the foci increased in size the following days. Passage of supernatant from transfected cells 8 d.p.t. onto new cells confirmed the presence of infectious particles, and packaging of genomes with the EGFP transgene into these (Figure 3b). CHSE cells infected with EGFPrSAV3 showed expression of EGFP already at 24 hours post infection (h.p.i.). The EGFPrSAV3 virus gave weak or no CPE compared to rSAV3, although the majority of cells were infected (EGFP positive). In one experiment, passage 3 of EGFPrSAV3 was grown in CHSE and BF2 cells for 30 days. After this prolonged incubation period less than 1% of cells still expressed EGFP, suggesting either gradual loss of the virus from cell culture or loss of/disruption of the transgene when incubation time is very long. During the work with constructing pEGFPrSAV3, a clone, pEGFPrSAV3_nsP3263M323G _was obtained that had accumulated mutations in nsP3 positions 263 (K to M) and 323 (E to G), likely a coincidence occurring during the cloning process. This clone was studied further in order to test the relative sensitivity of SAV to random mutations in this conserved region of nsP3. Although replicating and infectious recombinant virus expressing EGFP could be recovered after transfection (not shown), these clones appeared to spread slowly in infected cell cultures and was no longer detectable after two passages (Table 2).

**Table 2:** Detection of different SAVs in cell culture.

Virus	Transfection	Passage 1	Passage 2	Passage 3
SAVH20/03	+++	+++	+++	+++
rSAV3_E2206S_	ND	+++	+++	+++
rSAV3_E2206P_	ND	+++	+++	+++
rSAV3_nsP2197DE2206S_	+	+	(+)	-
rSAV3_nsP2197DE2206P_	+	+	+	-
EGFPrSAV _nsP3263M323G_	++	+	-	-
EGFPrSAV3	++	+++	+++	+++

BF2 cells were transfected with 5 μg of plasmid encoding the respective viruses, or infected with SAVH20/03. Supernatant was passed to new CHSE cells three times. All viruses were detected by IFAT using anti E2 antiserum, except EGFPrSAVnsP3mut and EGFPrSAV3 which were directly detected by fluorescence microscopy. The relative amount of positive cells for each virus is indicated (-/+/++/+++). SAVH20/03 served as positive control. ND = Not done.

### Mutations in nsP2_197 _and E2_206 _affect *in vitro *replication

**Figure 4:**
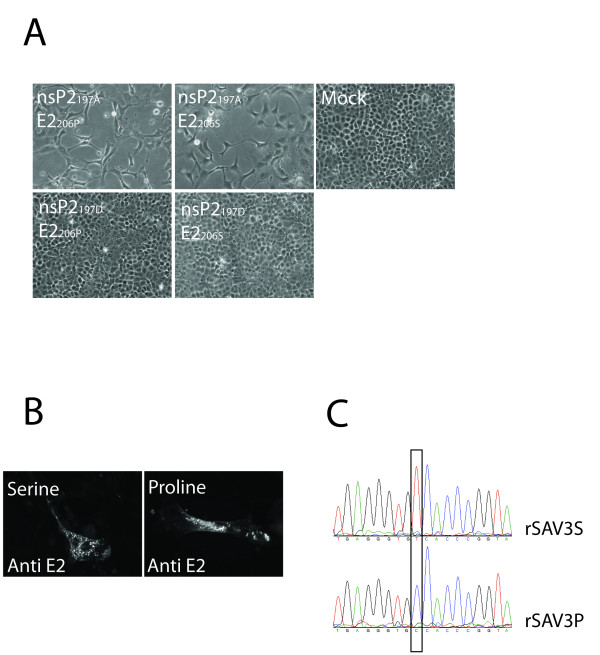
**Evaluation of mutations in nsP2 and E2**. The plasmid prSAV3 was modified to express either serine or proline in E2_206 _and either alanine or aspartic acid in nsP2_197_. (A) Infectious viruses were recovered from all plasmids after transfection into BF2 cells. The variants expressing the nsP2_197A _produced CPE in CHSE cells, while viruses expressing nsP2_197 D _did not. The E2_206 _mutation did not appear to affect CPE. Representative images of CPE are shown for each mutant. (B) The E2_206 _mutation did not affect antigenicity of the E2 protein notably, since both the serine and proline mutants were readily detected by IFAT. (C) RT-PCR and sequencing of passage 3 of recombinant viruses confirmed that both the serine and the proline codons were stable.

Previously, we have associated a serine to proline mutation in E2_206 _of SAVH20/03 with cell culture adaptation based on sequence comparison studies [25]. We also recently reported a replicon based on the SAVH20/03 genome to differ from the wild type sequence through an alanine to aspartic acid mutation in nsP2_197 _[9]. Through genetic modifications of the prSAV3 plasmid, the nsP2_197A _or nsP2_197 D _was expressed in combination with E2_206 S _or E2_206P _and all four viral mutants were recovered and shown to be infectious by serial passage in CHSE cells followed by IFAT (Table 2). A visible CPE indistinguishable from wild type CPE was produced by both mutants containing nsP2_197A_, while no CPE could be seen for mutants containing nsP2_197 D _(Figure 4a). IFAT on infected cells suggested that foci of infection were small with the nsP2_197 D _mutants and that the majority of cells remained uninfected. After three passages in cell culture, infection by the two rSAV3_nsP2197 D _virus variants could not be detected by IFAT, while cells infected with the clones containing the nsP2_197A _remained readily detectable (Table 2). The E2_S206P _mutation did not cause lack of recognition by the E2 antiserum since indistinguishable staining was obtained with the two virus mutants (Figure 4b). Sequencing of the partial E2 coding sequence verified that both these mutants were genetically stable during three passages in CHSE cells (Figure 4c). Endpoint titration of supernatant from infected cell layers confirmed that the virus titer was reduced in the nsP2_A197 D _mutant while for the E2_S206P _mutant the production of infectious particles appeared to increase by almost one log (Table 3). Recombinant SAV with E2_206P _produced similar titers as the wild type SAVH20/03 isolate also carrying the E2_206P_.

**Table 3:** Tissue culture infective dose (TCID50/ml) produced by wild type SAVH20/03 and different mutants 7 d.p.i. in CHSE cells.

Virus	TCID50/ml
SAVH20/03	1.6 × 10^7^
rSAV_E2206S_	2.8 × 10^6^
rSAV_E2206P_	1.6 × 10^7^
rSAV_nsP2197DE2206S_	< 10
rSAV_nsP2197DE2206P_	< 10

### The E2_P206 S _mutation has occurred recently and the position is located on the protein surface

**Figure 5:**
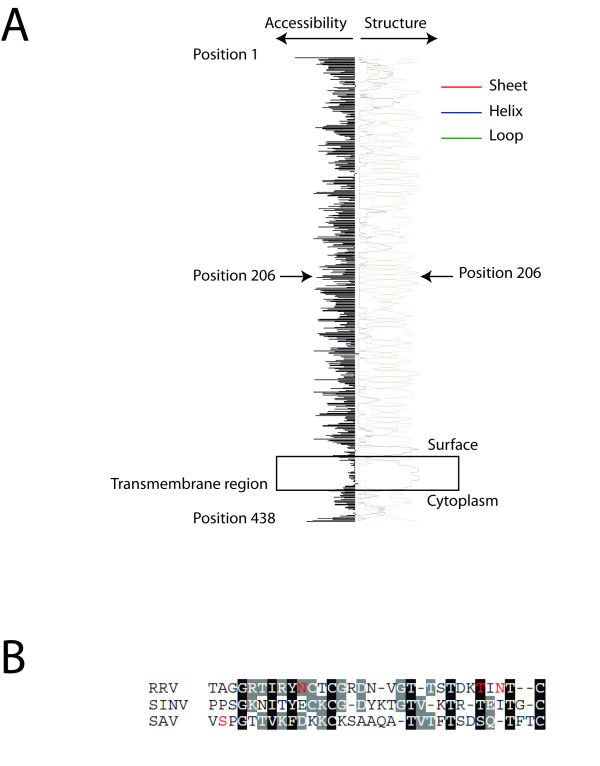
***In silico *analyses of E2 structure and accessibility**. (A) Secondary structure and solvent accessibility of the SAV3 E2 protein was estimated using the PredictProtein server and TMpred. Relative solvent accessibility is illustrated by black bars and predicted secondary structures are shown as red (sheet), green (loop) or blue (helix) lines. The predicted transmembrane helix is indicated. (B) Alignment of a partial SAV3 E2 sequence with a region of E2 from SINV and RRV previously suggested to be located on the protein surface. E2_206 _is indicated for SAV by red colour. E2_200_, E2_216 _and E2_218 _are indicated for RRV since they previously have been shown to be located on the very distal part of the spike [54].

**Figure 6:**
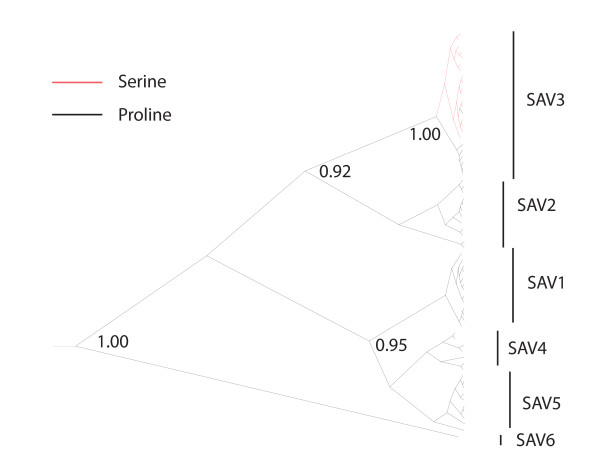
**Bayesian phylogenetic analysis of partial E2 sequences**. Clusters corresponding to the SAV1-6 subtypes are indicated. Sequences containing the E2_206 _serine are indicated by red branches, while black branches indicate proline. Posterior probabilities are given for key nodes.

Structural predictions of E2 using the PredictProtein server [36] and TMpred suggested that the transmembrane helix of SAVH20/03 E2 consists of amino acids E2_385A _to E2_404A_, while E2_206 _appears to be located in a loop area flanked by two sheet areas. Accessibility plots of the protein further predicted the E2_206 _position to be highly accessible (Figure 5a). Moreover, alignment with E2 from other alphaviruses supported the idea of SAV E2_206 _being located on the surface of the virus particle (Figure 5b). Finally, a reconstruction of the evolutionary history of the partial E2 protein of SAV suggested that the proline is the ancient aa in this position and that the serine has evolved in subtype 3 only (Figure 6). Some SAV3 isolates also still carry the proline.

## Discussion

SAV is a virus that is associated with mortality and significant economical loss for salmonid fish farmers in Europe [4]. Still, important questions related to viral and host mechanisms that contribute to transmission and pathogenesis remain unanswered. Here we report the development of infectious clones for a subtype 3 strain where the viral genome is transcribed by cellular RNA polymerase II from a CMV promoter in a pVAX1 backbone. The recombinant virus was also engineered to express non-viral RNA from an introduced, additional subgenomic promoter. Subtype 3 appears to be genetically homogenous, indicating a relatively recent common source for all known isolates [25]. We therefore designed the system to recover viruses that express proteins identical to those reported previously from consensus sequences of SAVH20/03, the only subtype 3 strain where both ORFs have been completely sequenced [9,18,25]. Such alphaviral reverse genetics systems are suitable for studies of evolution and mechanisms of emergence and pathogenesis. For CHIKV, development of infectious clones with high similarity to wild type CHIKV has led to identification of point-mutations important for the emergence of epidemic strains [32-34]. Using the obtained SAV3 infectious clones, we demonstrated that point-mutations indeed may alter the replication properties of SAV dramatically.

The infectious clones were generated with focus on expressing proteins that were identical in amino acid sequence to those previously reported from SAVH20/03. However, compared to previously reported sequences from SAVH20/03, the prSAV3 plasmid contains 10 mutations that do not lead to amino acid change (Table 1). Although such mutations not leading to amino acid changes certainly have the potential to affect viral replication, the obtained recombinant virus had titer production comparable to SAVH20/03 and other wild type SAV isolates in cell culture [37]. Similar to some other DNA-launched positive stranded RNA virus systems [38,39] the exact rSAV3 3'-end is uncertain since transcription is stopped by the BGH signal in the pVAX1 vector, leaving some nonsense (non-viral) sequence downstream of the introduced polyA tail. This nonsense sequence will most likely be lost during replication, since alphaviruses are believed to maintain and repair their polyadenylated 3'-ends by the use of their RC [40-42]. Still, future work could benefit from investigating the true 3'end of the obtained transcripts, and potentially developing a more authentic 3' end, for example by inserting a Hepatitis delta virus ribozyme immediately downstream of the polyA tail [39]. Such studies could also focus on understanding the impact of different lengths of polyA tails, as this has been reported to affect replication of alphaviruses [43].

Single point mutations in the RC have previously been shown to affect the replication efficiency of an attenuated strain of SAV2 [10]. When we recovered an infectious recombinant SAV3 strain, we made a similar observation, as a mutation in nsP2_197 _dramatically reduced the ability of the virus to infect and replicate in CHSE cells. This mutation is located in a conserved region of nsP2 in alphaviruses, believed to include an NTP binding motif that is essential for RNA triphosphatase, helicase and NTPase activity of the protein [44]. Thus, it is likely that an aspartic acid compared to the wild type alanine in this position reduces efficiency of the viral capping apparatus as well as helicase activity. Similarly, the virus with two mutations in nsP3 had a clearly reduced ability to replicate, and resulted in the loss of the virus from infected cells after two passages. Both mutations were located in a region of nsP3 that is conserved among alphaviruses, but outside the described macro domain [45,46]. Although these mutations demonstrate that SAV can tolerate single amino acid mutations in highly conserved motifs of the RC, they also address the relatively large impact such single mutations may have on the viral phenotype. This suggests that SAV, like other alphaviruses [31,32], has the potential of major changes in some phenotypic features as a result of minor genetic change. A similar observation has been done for a SAV2 strain which adapted to replication at an elevated temperature through six mutations in the structural ORF [10].

Recently, studies of CHIKV have shown the power of reverse genetics systems that are based on clones with genomic sequences similar to wild-type/field isolate sequences [31-33]. Such systems are suitable for identifying mutations that contribute to the emergence of outbreaks. Alphavirus epizootics appear to be particularly associated with point mutations in the viral receptor protein E2 or the fusion protein E1 [31,32]. These mutations could lead to changes in cell tropism and affinity for host species [32]. We utilized the rSAV3 system to confirm that the previously reported E2_S206P _mutation [25] enhances the viral titer production in CHSE cells. This is in agreement with the hypothesis that this mutation may be a beneficial adaptation for replication in CHSE cells. Our phylogenetic analyses suggested, however, that a proline is the ancient aa residue in this position of E2 in natural populations of SAV, and that the mutation towards serine is a relatively recent event that has happened in the SAV3 subtype only. Alignments of sequences from SAV3 isolates have previously demonstrated that several SAV3 isolates still carry the proline [17,25]. This suggests that a proline codon might still be part of a viral quasispecies in SAV3 strains. If so, it could indicate a regulator of pathogenesis. In further support of this idea, *in silico *analyses predicted the E2_206 _to be located on the surface of the protein, probably in an area used for receptor recognition. It appears, however, that the mutation does not have any major effect on the antigenic properties, since both the serine and proline mutants were readily detected by the antiserum that was raised against a short peptide containing this region. Clearly, data from experimental transmission studies in fish are needed in order to better understand the role, if any, of the E2_S206P _mutation during an *in vivo *infection.

The pEGFPrSAV3, having a second subgenomic alphavirus promoter, could be a potential tool for delivery and expression of foreign RNA in fish, e. g. an RNA coding for an antigen or a reporter protein. Like other alphavirus vectors, they could provide several advantages over traditional vaccine vectors, since expression of the antigen occurs in the context of a viral infection [47]. Another powerful future aspect of the pEGFPrSAV3 plasmid could be to address tissue tropism of SAV3 mutants through bioluminiscence imaging, as has been done for other alphaviruses [33,48,49]. Such studies could help clarify the role of tissue tropism in SAV pathogenesis. We observed, however, that rSAV3 with double subgenomic promoter is unstable similar to what has been shown with such constructs for other alphaviruses [50]. It is possible that the virus is able to sustain in only a small fraction of cells when it contains a transgene, but it is more likely that the transgene itself is gradually lost or mutated as the infection progresses due to the lack of selection pressure. Further development of these SAV based expression systems should therefore aim at incorporating selection pressure on the transgene, as has been done for SINV [51].

## Conclusion

We have developed a functional reverse genetics system that recovers recombinant SAV with identical proteins as those reported from the genetically distinct SAVH20/03 strain that was isolated in Norway in 2003 and suggested to represent a new viral subtype (SAV3) [18]. The system is suitable for studies of viral replication, evolution and pathogenesis. It may also be used as a gene delivery system in fish cells and for the development of live attenuated vaccines.

## Methods

### Cells and virus

Infected and non-infected Chinook salmon embryo (CHSE) and Bluegill fry (BF2) cells were grown as previously described [25]. SAVH20/03 was used to infect CHSE or BF2 cells that were incubated at 14°C. Cells used for transfection were grown to approximately 80% confluence prior to transfection. Transfected cells were incubated at 20°C for 24 h before transfer to 14°C due to increased effect of the CMV promoter at the elevated temperature.

### Development of Anti-E2 polyclonal antiserum

The coding sequence of the SAV3 E2 protein was aligned with known immunogenic motifs of alphaviruses SINV, RRV. Based on this alignment, a 348 bp cDNA fragment containing a partial coding sequence of the E2 protein, was amplified by RT-PCR from CHSE cells infected with SAVH20/03 using primers Fc1_E2 (CACCAAGGGCCACCACTTGTCCGA) and Rc1_E2 (TCACTTCACTTCCTTGCCTCCGC). The fragment was cloned into prokaryotic expression vector pET200/D-TOPO, to the C-terminus of a His-tag, using the Champion pET TOPO Directional Expression kit (Invitrogen) as recommended by the manufacturer. BL21 Star One Shot *E. coli *cells (Invitrogen) were transformed with the pET200/E2 plasmid and expression of the His-tagged peptide was induced using 1 mM isopropyl beta-D-thiogalacto-pyranoside (IPTG) for four hours. Expression was verified by SDS-PAGE gel and Western blot with the provided anti-his-G-HRP antibody. Purified and dialysed protein was used for immunization of a rabbit. The obtained anti-serum was pre-adsorbed onto monolayers of CHSE cells fixed with 4% paraformaldehyde in PBS and methanol.

### Indirect fluorescence antibody test (IFAT)

CHSE-214 or BF2 cells were grown on cover-slips in 24 well plates or in 96 well plates and infected with different SAV strains as indicated in the results section. The cells were fixed and permeabilized with 4% paraformaldehyde in PBS and methanol. The cells were then washed twice with PBS-FBS (PBS with 5% fetal bovine serum), before blocking using 5% skimmed milk in PBS-FBS for 30 min. The primary antibody anti-E2 was then diluted 1:400 and incubated on the cells in PBS-FBS with 5% skimmed milk for 60 min at room temperature. Following antibody incubation, cells were washed three times, and incubated with Alexa Fluor 488 goat anti-rabbit secondary antibody (Invitrogen) in PBS-FBS with 5% skimmed milk for 60 min at room temperature. Finally, the cells were washed three times with PBS and directly examined in an Olympus IXI fluorescence microscope, or mounted using ProLong Gold with DAPI (Invitrogen) and visualized using a Leica TCS SP2 AOBS confocal microscope. Mock infected cells and cells not treated with primary antibody served as negative controls.

### Construction of a full-length SAV3 cDNA clone

Total RNA was extracted from infected CHSE cells and reverse transcribed into cDNA using random hexamer primers according to standard techniques. Three PCR fragments, covering the entire structural ORF, were obtained and assembled by ligation into the pVAX1 vector (Invitrogen), leading to the pVAX1/SAV3 SP construct. The structural polyprotein ORF was further subcloned into a pCI vector (Promega) using the restriction sites EcoRI and NotI, after the correction of some mutations introduced during cloning, leading to the construct pCI/SAV3 SP*. In order to clone the 3'UTR sequence at the extremity of the SAV3 SP ORF sequence, the 3'UTR was obtained by RT-PCR using the primers PDuniF (CCGCACGGTTGTAAGATCAGT) and NotIXbaIPolyAR [9] and inserted into pCI/SAV3 SP* by StuI/NotI digestion and ligation, leading to the construct pCI/SAV3 SP+3'UTR.

An XbaI site was introduced by site-directed mutagenesis into the junction region within a previously reported replicon construct that contains a SAV3 replicon expressing EGFP in a pVAX1 backbone [9]. The construct was further engineered to express the wild type nsP2_197A_, leading to the plasmid pmSAV3wt. The EGFP ORF and 3'UTR was then exchanged with an XbaI fragment from the pCI/SAV3 SP+3'UTR plasmid. The authenticity of the obtained full-length construct, prSAV3, was verified by REN analysis and DNA sequencing as previously described [25]. All plasmids were replicated in XL10 Gold competent *E. coli *cells (Stratagene), and purified using Qiagen Endofree plasmid maxi kit according to the manufacturer's protocol.

### Recovery of infectious recombinant virus

The prSAV3 plasmid (5 μg) was transfected into BF2 cells (passage 0) using Amaxa nucleofector kit T (Lonza) as recommended by the manufacturer. Transfected cells were then cultured as previously described [9]. Twelve d.p.t., supernatant from transfected cells was passed to freshly prepared CHSE cells. Transfected and infected cell layers were tested by IFAT for expression of the E2 glycoprotein. Infected CHSE cells (passage 3) were harvested 7 days p.i., and extracted RNA was used as template for RT-PCR using SAV3 specific primers 544F (ATGATATGATGGTGGCCAGG) and 1238R (GCTCCCTTTCTTCTCCTGTTG). The obtained PCR product was sequenced and analysed for the presence of the introduced XbaI site.

### Construction and recovery of recombinant SAV3 carrying an additional subgenomic transcription cassette

A plasmid pEGFPrSAV3 where an additional ORF encoding EGFP under the SAV3 subgenomic promoter is placed upstream of the SAV3 structural transcription unit, was constructed based on the pmSAV3wt plasmid. A cDNA containing the structural ORF, 3' UTR, polyA tail and a presumed SAV3 subgenomic promoter which includes the junction region and the 3'-terminal 92 nucleotides of nsP4 (including the stop codon), was amplified from the prSAV3 plasmid using primers nsP4pro (GGGGCGCGCCGCGTGAATCACCTGCCGTTAGCCAC) and NotIXbaIPolyAR. The obtained cDNA was inserted into pmSAV3wt by AscI/NotI digestion and ligation. Authenticity of the plasmid was verified by sequencing of the complete transcription unit. One of the obtained clones, named pEGFPrSAV3_nsP3263M323G_, contained two amino acid mutations in nsP3 that probably were introduced as artefacts during cloning. Transfection and recovery of infectious virus was done as described above, and expression of the EGFP transgene was verified by direct fluorescence microscopy of infected cells and photographed using an Olympus IXI camera.

### Analysis of the effect of point mutations in nsP2 and E2 on the viral titer production

Infectious clones were engineered to test the effect of the nsP2_D197A _and E2_S206P _mutations. The clone prSAV3_E2206P _was made by site-directed mutagenesis using a QuickChange XL Mutagenesis kit (Stratagene). The clones prSAV3_nsP2197DE2206 S _and prSAV3 _nsP2197DE2206P _were generated by replacing the BstBI/NhiI fragments in prSAV3 and prSAV3_E2206P _with one from the previously reported pmSAV3 plasmid. The transcription units of all plasmids were sequenced completely prior to transfection. Transfections and recovery of infectious virus was done as described above. Recovered viruses were passaged in CHSE cells and supernatant from day 7 p.i. was titrated in 96 well plates using endpoint titration. Titers were calculated using the Kärber formula [52]. In addition to cytopathic effect, IFAT with anti-E2 was done in all wells as described above in order to confirm the presence or absence of infection. Finally, a cDNA containing the partial E2 gene was amplified by RT-PCR using primers F2234 and SAV20R [25] and sequenced from infected cell layers in passage three in order to verify that the E2_206P _and E2_206 S _mutations were stable.

### Bioinformatical analyses

Predictions of protein secondary structure and solvent accessibility were obtained by submitting the complete E2 sequence of SAVH20/03 to the PredictProtein server [35]. Transmembrane helices were predicted using TMpred http://www.ch.embnet.org/software/TMPRED_form.html. A nucleotide alignment (357 nt) was generated based on 67 previously reported SAV sequences covering the E2 position 206 (accession numbers AJ316244, AJ316246, EF675551 to EF675577, EF675579 to EF675594 and DQ122127 to DQ122146) [17,25]. Sequences were aligned by AlignX in the VectorNTI package (Invitrogen). The alignment was imported into the software package BEAST for Bayesian reconstruction of phylogeny [53]. The GTR model of nucleotide evolution with a four category gamma rate, and a constant population size assumption, was chosen. Trees were constructed using a relaxed lognormal clock assumption. A prior in lognormal distribution was set on the mean rate parameter, based on a previously reported substitution rate [25]. The MCMC chains were run for 20 million generations and the results were inspected in Tracer 1.4. ESS values were higher than 200 for all parameters. A maximum clade credibility tree was viewed using FigTree, and the tree was obtained using a 10% burnin in TreeAnnotator.

## Competing interests

The authors declare that they have no competing interests.

## Authors' contributions

MK planned the study, conducted all laboratory and bioinformatical work except production of E2 antiserum and cloning of the structural polyprotein ORF, analysed the results and wrote the manuscript. SV contributed to experimental design, performed the cloning of the structural polyprotein ORF and critically revised the manuscript. KFO developed the E2 antiserum and contributed to the revision of the manuscript. ER has contributed with discussions throughout the study and reading and contributing to the writing of the manuscript. AN has contributed during planning and with discussions throughout the study. All authors read and approved the final manuscript.

## References

[B1] Strauss JH Strauss EG The alphaviruses: gene expression, replication, and evolution Microbiol Rev 1994 58 3 491 562 7968923 10.1128/mr.58.3.491-562.1994 PMC372977

[B2] Weston JH Welsh MD McLoughlin MF Todd D Salmon pancreas disease virus, an alphavirus infecting farmed Atlantic salmon, Salmo salar L Virology 1999 256 2 188 195 10.1006/viro.1999.9654 10191183

[B3] Villoing S Bearzotti M Chilmonczyk S Castric J Bremont M Rainbow trout sleeping disease virus is an atypical alphavirus J Virol 2000 74 1 173 183 10.1128/JVI.74.1.173-183.2000 10590104 PMC111526

[B4] McLoughlin MF Graham DA Alphavirus infections in salmonids--a review J Fish Dis 2007 30 9 511 531 10.1111/j.1365-2761.2007.00848.x 17718707

[B5] Weston J Villoing S Bremont M Castric J Pfeffer M Jewhurst V McLoughlin M Rodseth O Christie KE Koumans J Comparison of two aquatic alphaviruses, salmon pancreas disease virus and sleeping disease virus, by using genome sequence analysis, monoclonal reactivity, and cross-infection J Virol 2002 76 12 6155 6163 10.1128/JVI.76.12.6155-6163.2002 12021349 PMC136221

[B6] Firth AE Chung BY Fleeton MN Atkins JF Discovery of frameshifting in Alphavirus 6K resolves a 20-year enigma Virol J 2008 5 108 10.1186/1743-422X-5-108 18822126 PMC2569925

[B7] Moriette C LeBerre M Boscher SK Castric J Bremont M Characterization and mapping of monoclonal antibodies against the Sleeping disease virus, an aquatic alphavirus J Gen Virol 2005 86 Pt 11 3119 3127 10.1099/vir.0.81030-0 16227235

[B8] Karlsen M Yousaf MN Villoing S Nylund A Rimstad E The amino terminus of the salmonid alphavirus capsid protein determines subcellular localization and inhibits cellular proliferation Arch Virol 2010 155 8 1281 1293 10.1007/s00705-010-0717-x 20556445

[B9] Karlsen M Villoing S Rimstad E Nylund A Characterization of untranslated regions of the salmonid alphavirus 3 (SAV3) genome and construction of a SAV3 based replicon Virol J 2009 6 173 10.1186/1743-422X-6-173 19860871 PMC2772843

[B10] Moriette C Leberre M Lamoureux A Lai TL Bremont M Recovery of a recombinant salmonid alphavirus fully attenuated and protective for rainbow trout J Virol 2006 80 8 4088 4098 10.1128/JVI.80.8.4088-4098.2006 16571825 PMC1440445

[B11] Garmashova N Gorchakov R Volkova E Paessler S Frolova E Frolov I The Old World and New World alphaviruses use different virus-specific proteins for induction of transcriptional shutoff J Virol 2007 81 5 2472 2484 10.1128/JVI.02073-06 17108023 PMC1865960

[B12] Elgizoli M Dai Y Kempf C Koblet H Michel MR Semliki Forest virus capsid protein acts as a pleiotropic regulator of host cellular protein synthesis J Virol 1989 63 7 2921 2928 2724418 10.1128/jvi.63.7.2921-2928.1989 PMC250845

[B13] Favre D Studer E Michel MR Two nucleolar targeting signals present in the N-terminal part of Semliki Forest virus capsid protein Arch Virol 1994 137 1-2 149 155 10.1007/BF01311181 7979988

[B14] Michel MR Elgizoli M Dai Y Jakob R Koblet H Arrigo AP Karyophilic properties of Semliki Forest virus nucleocapsid protein J Virol 1990 64 10 5123 5131 2398536 10.1128/jvi.64.10.5123-5131.1990 PMC248004

[B15] Mitchell C de Andrade-Rozental AF Souto-Padron T Carvalho MG Identification of mayaro virus nucleocapsid protein in nucleus of Aedes albopictus cells Virus Res 1997 47 1 67 77 10.1016/S0168-1702(96)01408-6 9037738

[B16] Garoff H Sjoberg M Cheng RH Budding of alphaviruses Virus Res 2004 106 2 103 116 10.1016/j.virusres.2004.08.008 15567491

[B17] Fringuelli E Rowley HM Wilson JC Hunter R Rodger H Graham DA Phylogenetic analyses and molecular epidemiology of European salmonid alphaviruses (SAV) based on partial E2 and nsP3 gene nucleotide sequences J Fish Dis 2008 31 11 811 823 10.1111/j.1365-2761.2008.00944.x 18681902

[B18] Hodneland K Bratland A Christie KE Endresen C Nylund A New subtype of salmonid alphavirus (SAV), Togaviridae, from Atlantic salmon Salmo salar and rainbow trout Oncorhynchus mykiss in Norway Dis Aquat Organ 2005 66 2 113 120 10.3354/dao066113 16231636

[B19] Graham DA Rowley HM Walker IW Weston JH Branson EJ Todd D First isolation of sleeping disease virus from rainbow trout, Oncorhynchus mykiss (Walbaum), in the United Kingdom J Fish Dis 2003 26 11-12 691 694 10.1046/j.1365-2761.2003.00505.x 14710763

[B20] Andersen L Bratland A Hodneland K Nylund A Tissue tropism of salmonid alphaviruses (subtypes SAV1 and SAV3) in experimentally challenged Atlantic salmon (Salmo salar L.) Arch Virol 2007 152 10 1871 1883 10.1007/s00705-007-1006-1 17578649

[B21] Christie KE Graham DA McLoughlin MF Villoing S Todd D Knappskog D Experimental infection of Atlantic salmon Salmo salar pre-smolts by i.p. injection with new Irish and Norwegian salmonid alphavirus (SAV) isolates: a comparative study Dis Aquat Organ 2007 75 1 13 22 10.3354/dao075013 17523539

[B22] Boscher SK McLoughlin M Le Ven A Cabon J Baud M Castric J Experimental transmission of sleeping disease in one-year-old rainbow trout, Oncorhynchus mykiss (Walbaum), induced by sleeping disease virus J Fish Dis 2006 29 5 263 273 10.1111/j.1365-2761.2006.00716.x 16677316

[B23] Taksdal T Olsen AB Bjerkas I Hjortaas MJ Dannevig BH Graham DA McLoughlin MF Pancreas disease in farmed Atlantic salmon, Salmo salar L., and rainbow trout, Oncorhynchus mykiss (Walbaum), in Norway J Fish Dis 2007 30 9 545 558 10.1111/j.1365-2761.2007.00845.x 17718709

[B24] Crockford T Menzies FD McLoughlin MF Wheatley SB Goodall EA Aspects of the epizootiology of pancreas disease in farmed Atlantic salmon *Salmo salar *in Ireland Dis Aquat Organ 1999 36 113 119 10.3354/dao036113 10399039

[B25] Karlsen M Hodneland K Endresen C Nylund A Genetic stability within the Norwegian subtype of salmonid alphavirus (family Togaviridae) Arch Virol 2006 151 5 861 874 10.1007/s00705-005-0687-6 16362641

[B26] Bratland A Nylund A Studies on the possibility of vertical transmission of Norwegian salmonid Alphavirus in production of Atlantic salmon in Norway J Aquat Anim Health 2009 21 3 173 178 10.1577/H08-038.1 20043403

[B27] Kristoffersen AB Viljugrein H Kongtorp RT Brun E Jansen PA Risk factors for pancreas disease (PD) outbreaks in farmed Atlantic salmon and rainbow trout in Norway during 2003-2007 Prev Vet Med 2009 90 1-2 127 136 10.1016/j.prevetmed.2009.04.003 19419787

[B28] Viljugrein H Staalstrom A Molvaelr J Urke HA Jansen PA Integration of hydrodynamics into a statistical model on the spread of pancreas disease (PD) in salmon farming Dis Aquat Organ 2009 88 1 35 44 10.3354/dao02151 20183963

[B29] Graham DA Jewhurst H McLoughlin MF Sourd P Rowley HM Taylor C Todd D Sub-clinical infection of farmed Atlantic salmon Salmo salar with salmonid alphavirus--a prospective longitudinal study Dis Aquat Organ 2006 72 3 193 199 10.3354/dao072193 17190198

[B30] Graham DA Fringuelli E Wilson C Rowley HM Brown A Rodger H McLoughlin MF McManus C Casey E McCarthy LJ Prospective longitudinal studies of salmonid alphavirus infections on two Atlantic salmon farms in Ireland; evidence for viral persistence J Fish Dis 33 2 123 135 10.1111/j.1365-2761.2009.01096.x 19732268

[B31] Anishchenko M Bowen RA Paessler S Austgen L Greene IP Weaver SC Venezuelan encephalitis emergence mediated by a phylogenetically predicted viral mutation Proc Natl Acad Sci USA 2006 103 13 4994 4999 10.1073/pnas.0509961103 16549790 PMC1458783

[B32] Tsetsarkin KA Vanlandingham DL McGee CE Higgs S A single mutation in chikungunya virus affects vector specificity and epidemic potential PLoS Pathog 2007 3 12 e201 10.1371/journal.ppat.0030201 18069894 PMC2134949

[B33] Tsetsarkin K Higgs S McGee CE De Lamballerie X Charrel RN Vanlandingham DL Infectious clones of Chikungunya virus (La Reunion isolate) for vector competence studies Vector Borne Zoonotic Dis 2006 6 4 325 337 10.1089/vbz.2006.6.325 17187566

[B34] Tsetsarkin KA McGee CE Volk SM Vanlandingham DL Weaver SC Higgs S Epistatic roles of E2 glycoprotein mutations in adaption of chikungunya virus to Aedes albopictus and Ae. aegypti mosquitoes PLoS One 2009 4 8 e6835 10.1371/journal.pone.0006835 19718263 PMC2729410

[B35] Xu C Guo TC Mutoloki S Haugland Ø Marjara IS Evensen Ø Alpha interferon and not gamma interferon inhibits salmonid alphavirus subtype 3 replication in vitro J Virol 2010 84 17 8903 8912 10.1128/JVI.00851-10 20573808 PMC2919011

[B36] Rost B Yachdav G Liu J The PredictProtein server Nucleic Acids Res 2004 32 Web Server W321 326 10.1093/nar/gkh377 15215403 PMC441515

[B37] Graham DA Wilson C Jewhurst H Rowley H Cultural characteristics of salmonid alphaviruses--influence of cell line and temperature J Fish Dis 2008 31 11 859 868 10.1111/j.1365-2761.2008.00946.x 19238759

[B38] Lee C Calvert JG Welch SK Yoo D A DNA-launched reverse genetics system for porcine reproductive and respiratory syndrome virus reveals that homodimerization of the nucleocapsid protein is essential for virus infectivity Virology 2005 331 1 47 62 10.1016/j.virol.2004.10.026 15582652

[B39] Dubensky TW Driver DA Polo JM Belli BA Latham EM Ibanez CE Chada S Brumm D Banks TA Mento SJ Sindbis virus DNA-based expression vectors: utility for in vitro and in vivo gene transfer J Virol 1996 70 1 508 519 8523564 10.1128/jvi.70.1.508-519.1996 PMC189839

[B40] Hardy RW The role of the 3' terminus of the Sindbis virus genome in minus-strand initiation site selection Virology 2006 345 2 520 31 10.1016/j.virol.2005.10.018 16297426

[B41] Tomar S Hardy RW Smith JL Kuhn RJ Catalytic core of alphavirus nonstructural protein nsP4 possesses terminal adenylyltransferase activity J Virol 2006 80 20 9962 9969 10.1128/JVI.01067-06 17005674 PMC1617302

[B42] Raju R Hajjou M Hill KR Botta V Botta S In vivo addition of poly(A) tail and AU-rich sequences to the 3' terminus of the Sindbis virus RNA genome: a novel 3'-end repair pathway J Virol 1999 73 3 2410 2419 9971825 10.1128/jvi.73.3.2410-2419.1999 PMC104487

[B43] Hardy RW Rice CM Requirements at the 3' end of the sindbis virus genome for efficient synthesis of minus-strand RNA J Virol 2005 79 8 4630 4639 10.1128/JVI.79.8.4630-4639.2005 15795249 PMC1069581

[B44] Vasiljeva L Merits A Auvinen P Kaariainen L Identification of a novel function of the alphavirus capping apparatus. RNA 5'-triphosphatase activity of Nsp2 J Biol Chem 2000 275 23 17281 17287 10.1074/jbc.M910340199 10748213

[B45] Gorbalenya AE Koonin EV Lai MM Putative papain-related thiol proteases of positive-strand RNA viruses. Identification of rubi- and aphthovirus proteases and delineation of a novel conserved domain associated with proteases of rubi-, alpha- and coronaviruses FEBS Lett 1991 288 1-2 201 205 10.1016/0014-5793(91)81034-6 1652473 PMC7130274

[B46] Malet H Coutard B Jamal S Dutartre H Papageorgiou N Neuvonen M Ahola T Forrester N Gould EA Lafitte D The crystal structures of Chikungunya and Venezuelan equine encephalitis virus nsP3 macro domains define a conserved adenosine binding pocket J Virol 2009 83 13 6534 6545 10.1128/JVI.00189-09 19386706 PMC2698539

[B47] Schlesinger S Alphavirus expression vectors Adv Virus Res 2000 55 565 577 full_text 11050957 10.1016/s0065-3527(00)55018-0

[B48] Morrison TE Whitmore AC Shabman RS Lidbury BA Mahalingam S Heise MT Characterization of Ross River virus tropism and virus-induced inflammation in a mouse model of viral arthritis and myositis J Virol 2006 80 2 737 749 10.1128/JVI.80.2.737-749.2006 16378976 PMC1346871

[B49] Vanlandingham DL Tsetsarkin K Hong C Klingler K McElroy KL Lehane MJ Higgs S Development and characterization of a double subgenomic chikungunya virus infectious clone to express heterologous genes in Aedes aegypti mosquitoes Insect Biochem Mol Biol 2005 35 10 1162 1170 10.1016/j.ibmb.2005.05.008 16102421

[B50] Rausalu K Iofik A Ulper L Karo-Astover L Lulla V Merits A Properties and use of novel replication-competent vectors based on Semliki Forest virus Virol J 2009 6 33 10.1186/1743-422X-6-33 19317912 PMC2669057

[B51] Thomas JM Klimstra WB Ryman KD Heidner HW Sindbis virus vectors designed to express a foreign protein as a cleavable component of the viral structural polyprotein J Virol 2003 77 10 5598 5606 10.1128/JVI.77.10.5598-5606.2003 12719552 PMC154044

[B52] WHO Manual for the virological investigation of polio Geneva 1997

[B53] Drummond AJ Rambaut A BEAST: Bayesian evolutionary analysis by sampling trees BMC Evol Biol 2007 7 214 10.1186/1471-2148-7-214 17996036 PMC2247476

[B54] Zhang W Heil M Kuhn RJ Baker TS Heparin binding sites on Ross River virus revealed by electron cryo-microscopy Virology 2005 332 2 511 518 10.1016/j.virol.2004.11.043 15680416 PMC4152768

